# Supplementary figures and images for: Running behaviors, motivations, and injury risk during the COVID-19 pandemic: A survey of 1147 runners
Source: PLoS One. 2021 Feb 12;16(2):e0246300. doi: 10.1371/journal.pone.0246300 (PMC7880469; doi:10.1371/journal.pone.0246300)

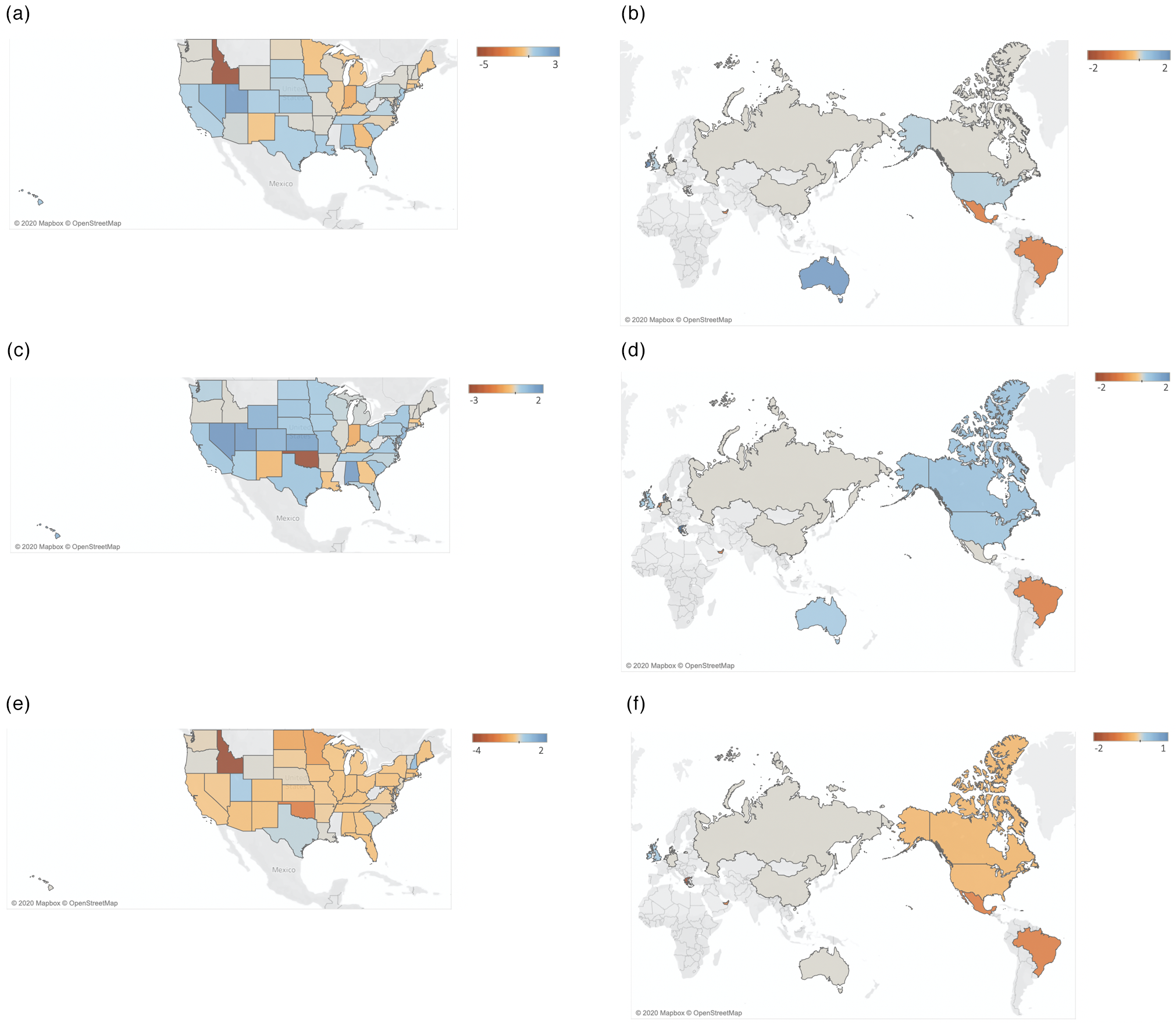

Supplement: S1 Fig — Abbreviations: Avg, Average; Diff, Difference; N, number. (TIF) [file pone.0246300.s001.tif]
