# Supplementary material for: Running behaviors, motivations, and injury risk during the COVID-19 pandemic: A survey of 1147 runners
Source: PLoS One. 2021 Feb 12;16(2):e0246300. doi: 10.1371/journal.pone.0246300 (PMC7880469; doi:10.1371/journal.pone.0246300)
Supplement: S1 File — List of questions and response options included in the survey. Question logic was used to skip irrelevant questions and displayed within the document. (DOCX) [file pone.0246300.s002.docx]

Running Behaviors During COVID-19

Start of Block: Consent Form

**Study Information Sheet. Please read this study information sheet carefully before you decide to participate in the study. Study Title:**Running Behaviors during the COVID-19 Pandemic. **Purpose of the research study:** The purpose of this study is to assess the effects of the COVID-19 pandemic on running behaviors among runners 18 and older, with an emphasis on training volume, motives for running, and running-related injury pre-, during, and post-social isolation. **What you will do in the study:** You will be asked to fill out information on your age, gender, geographical location, running patterns, and running-related injury status in this survey. You will also be asked to complete a follow-up survey at a later date to assess your running behaviors transitioning back after the pandemic, and we will ask you to please provide an email address for this purpose. **Time required:** The first survey will require about 10-15 minutes of your time, and the follow-up survey to be sent at a later date will take approximately 5-10 minutes of your time. **Risks:** There are no risks of participating in this study. **Benefits:** There are no direct benefits to you for participating in this research study.  The study may help us understand how runners are adapting to the change imposed by the COVID-19 pandemic, and get some insight on the injury patterns during this time. **Confidentiality:** The information that you give in the study will be handled confidentially.  Your information will be assigned a code number.  The list connecting your email address to this code will be kept in a locked file.  When the study is completed and the data have been analyzed, this list will be destroyed.  Your name will not be used in any report. **Voluntary participation:** Your participation in the study is completely voluntary.  **Right to withdraw from the study:** You have the right to withdraw from the study at any time without penalty.  **How to withdraw from the study:** You may stop completing the survey at any time by closing out of the browser. There is no penalty for withdrawing. **Payment:** You will receive no payment for participating in the study. **Using data beyond this study:**The data you provide in this study will be retained in a secure manner by the researcher for 2 years and then destroyed. **If you have questions about the study, contact:**Alexandra DeJong (Study Coordinator)
Department of Kinesiology, 210 Emmet Street S
University of Virginia, Charlottesville, VA 22903.  
Telephone: (434) 924-6184
afd4au@virginia.eduDr. Jay Hertel (PI)
Department of Kinesiology, 210 Emmet Street S
University of Virginia, Charlottesville, VA 22903.  
Telephone: (434) 243-8673
jnh7g@virginia.edu

**To obtain more information about the study, ask questions about the research procedures, express concerns about your participation, or report illness, injury or other problems, please contact:**
Tonya R. Moon, Ph.D.
Chair, Institutional Review Board for the Social and Behavioral Sciences
One Morton Dr Suite 500 
University of Virginia, P.O. Box 800392
Charlottesville, VA 22908-0392
Telephone:  (434) 924-5999 
Email: irbsbshelp@virginia.edu
Website: https://research.virginia.edu/irb-sbs
Website for Research Participants: https://research.virginia.edu/research-participantsUVA IRB-SBS # 3677If you agree to participate, press YES. **You may print a copy of this document for your records.**

- Yes, I consent to participate (1)
- No, I do not consent to participate (2)

Skip To: End of Survey If Study Information Sheet Please read this study information sheet carefully before you decide to p... = No, I do not consent to participate

End of Block: Consent Form

Start of Block: Demographics

Q3 What is your age (in years)?

▼ 18 (9) ... Over 80 years of age (72)

Q1 How long have you been running regularly (in years and months)

▼ less than 1 month (1) ... 20 or more years (19)

Q4 What is your biological sex?

- Male (1)
- Female (2)
- Prefer not to answer (3)

Q6 Do you currently live in the United States?

- Yes (1)
- No (2)

Skip To: Q8 If Do you currently live in the United States? = Yes

Display This Question:

If Do you currently live in the United States? = No

Q9 What country do you currently live in?

________________________________________________________________

Display This Question:

If Do you currently live in the United States? = Yes

Q8 Which state do you currently live in?

▼ Alabama (7) ... Wyoming (56)

End of Block: Demographics

Start of Block: Running behaviors in the last year prior to the COVID-19 pandemic

Q43 Please respond to the next set of questions in regards to your running behaviors***in the year prior to social distancing restrictions in your region due to the COVID-19 pandemic***

| Page Break |  |
| --- | --- |

Q10 **In the year prior to social distancing restrictions in your region due to the COVID-19 pandemic,**how many times per week did you perform:

|  | 0 | 1 | 2 | 3 | 4 | 5 | 6 | 7 | 8 | 9 | 10 | 11 | 12 | 13 | 14 |
| --- | --- | --- | --- | --- | --- | --- | --- | --- | --- | --- | --- | --- | --- | --- | --- |

| All Runs () | 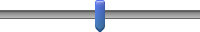 |
| --- | --- |
| Sustained Runs () | 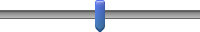 |
| Workouts (i.e. track intervals, fartleks) () | 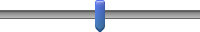 |
| Cross-Training Activities (i.e. yoga, strength training, cycling, swimming,...) () | 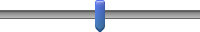 |

Q26 **In the year prior to social distancing restrictions in your region due to the COVID-19 pandemic,** what was your typical running pace (minutes/miles) for:

|  |  |
| --- | --- |
| Sustained Runs (77) | ▼ N/A (88) ... 14:00 (86) |
| Workouts (ie. fartleks, intervals, speed training) (78) | ▼ N/A (88) ... 14:00 (86) |

Q11 How many miles per week on average did you run**in the year prior to social distancing restrictions in your region due to the COVID-19 pandemic?**

▼ 0 (104) ... greater than 120 (25)

Q15 **In the year prior to social distancing restrictions in your region due to the COVID-19 pandemic,** what were your motives for running (select all that apply):

- Exercise/Fitness (1)
- Competition/Races (2)
- Socialization (3)
- Stress Relief (4)
- Enjoyment/Pleasure (5)
- Occupy Free Time (6)

Q23 **In the year prior to social distancing restrictions in your region due to the COVID-19 pandemic,** did you primarily perform runs indoors, outdoors, or both?

- Indoors (1)
- Outdoors (2)
- Both (4)

Q24 **In the year prior to social distancing restrictions in your region due to the COVID-19 pandemic,** did you primarily perform runs alone, in groups, or both?

- Alone (1)
- Groups (2)
- Both (4)

Q25 **In the year prior to social distancing restrictions in your region due to the COVID-19 pandemic,** did you use any form of technology to track your runs (i.e. smart watch, running phone application)?

- Yes (1)
- No (2)

Q27 **In the year prior to social distancing restrictions in your region due to the COVID-19 pandemic,** what time of day did you typically run (select all that apply)?

- Early morning (5am-7am) (1)
- Mid-Morning (8am-10am) (2)
- Midday (11am-1pm) (3)
- Early Afternoon (2pm-4pm) (4)
- Afternoon (5pm-7pm) (5)
- Evening (8pm-10pm) (6)
- Night Runs (11pm-4am) (7)

End of Block: Running behaviors in the last year prior to the COVID-19 pandemic

Start of Block: Injury History Prior to COVID-19

Q19 **In the year prior to social distancing restrictions in your region due to the COVID-19 pandemic,** did you suffer any running-related injuries?

- Yes (1)
- No (2)

Skip To: End of Block If In the year prior to social distancing restrictions in your region due to the COVID-19 pandemic,... = No

Q40 How many running-related injuries did you sustain **in the year prior to social distancing restrictions in your region due to the COVID-19 pandemic?**

▼ 1 (1) ... Greater than 10 (11)

Q42 How long were you unable to run due to running-related injuries sustained **in the year prior to social distancing restrictions in your region due to the COVID-19 pandemic?**

▼ 1 day (1) ... 11-12 months (16)

Q33 How long did you have to modify your running training due to running-related injuries sustained **in the year prior to social distancing restrictions in your region due to the COVID-19 pandemic?**

▼ 1 day (1) ... 11-12 months (54)

Q23 For the running-related injuries you sustained **in the year prior to social distancing restrictions in your region due to the COVID-19 pandemic**, please type the number of injury types occurring at each body location in the text boxes provided.

|  | Injury Type | | | |
| --- | --- | --- | --- | --- |
|  | Sprain (ligament) (1) | Strain (muscle or tendon) (2) | Fracture (broken bone) (3) | Other (please explain) (4) |
| Toe (1) |  |  |  |  |
| Foot (10) |  |  |  |  |
| Ankle (2) |  |  |  |  |
| Lower leg (3) |  |  |  |  |
| Knee (4) |  |  |  |  |
| Thigh (5) |  |  |  |  |
| Hamstring (6) |  |  |  |  |
| Hip (7) |  |  |  |  |
| Groin (11) |  |  |  |  |
| Abdomen (8) |  |  |  |  |
| Low Back (9) |  |  |  |  |

End of Block: Injury History Prior to COVID-19

Start of Block: Running behaviors during the COVID-19 pandemic

Q44 Please respond to the next set of questions in regards to your running behaviors ***during social distancing restrictions in your region due to the COVID-19 pandemic.***

| Page Break |  |
| --- | --- |

Q30 **During social distancing restrictions in your region due to the COVID-19 pandemic,** how many times per week did you perform:

|  | 0 | 1 | 2 | 3 | 4 | 5 | 6 | 7 | 8 | 9 | 10 | 11 | 12 | 13 | 14 |
| --- | --- | --- | --- | --- | --- | --- | --- | --- | --- | --- | --- | --- | --- | --- | --- |

| All Runs () | 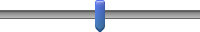 |
| --- | --- |
| Sustained Runs () | 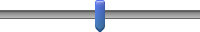 |
| Workouts (I.e. track intervals, fartleks) () | 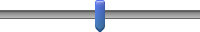 |
| Cross-Training Activities (i.e. yoga, strength training, cycling, swimming,...) () | 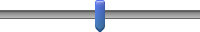 |

Q42 **During social distancing restrictions in your region due to the COVID-19 pandemic,** what was your typical running pace (minutes/miles) for:

|  |  |
| --- | --- |
| Sustained Runs (77) | ▼ N/A (88) ... 14:00 (86) |
| Workouts (ie. fartleks, intervals, speed training) (78) | ▼ N/A (88) ... 14:00 (86) |

Q40 How many miles per week on average did you run**during social distancing restrictions in your region due to the COVID-19 pandemic?**

▼ 0 (104) ... greater than 120 (25)

Q37 Has your mileage been different than expected around this current timeframe in your training plan?

- Yes (1)
- No (2)
- Not applicable to me (3)

Q32 **During social distancing restrictions in your region due to the COVID-19 pandemic,** what were your motives for running (select all that apply):

- Exercise/Fitness (1)
- Competition/Races (2)
- Socialization (3)
- Stress Relief (4)
- Enjoyment/Pleasure (5)
- Occupy Free Time (6)

Q31 **During social distancing restrictions in your region due to the COVID-19 pandemic,** did you primarily perform runs indoors, outdoors, or both?

- Indoors (1)
- Outdoors (2)
- Both (4)

Q36 Has your training environment changed due to social distancing restrictions in your region (i.e. moving, access to trails, paths, gyms)

- Yes (1)
- No (2)

Q29 **During social distancing restrictions in your region due to the COVID-19 pandemic,**did you primarily perform runs alone, in groups, or both?

- Alone (1)
- Groups (2)
- Both (4)

Q37 **During social distancing restrictions in your region due to the COVID-19 pandemic,** did you use any form of technology to track your runs (i.e. smart watch, running phone application)?

- Yes (1)
- No (2)

Q33 **During social distancing restrictions in your region due to the COVID-19 pandemic,** what time of day did you typically run? (select all that apply)

- Early morning (5am-7am) (1)
- Mid-Morning (8am-10am) (2)
- Midday (11am-1pm) (3)
- Early Afternoon (2pm-4pm) (4)
- Afternoon (5pm-7pm) (5)
- Evening (8pm-10pm) (6)
- Night Runs (11pm-4am) (7)

Q21 How much would you say your running training has changed **during social distancing restrictions in your region due to the COVID-19 pandemic?**

- It has increased a great deal (1)
- It has increased a lot (2)
- It has increased a moderate amount (3)
- It has increased a little (4)
- No change at all (5)
- It has decreased a little (6)
- It has decreased a moderate amount (7)
- It has decreased a lot (8)
- It has decreased a great deal (9)

Q22 How concerned are you about how the COVID-19 pandemic is **currently** affecting your running training?

- Very concerned (1)
- Somewhat concerned (2)
- Neutral (3)
- Somewhat unconcerned (4)
- Not concerned at all (5)

Q43 How concerned are you about how the COVID-19 pandemic is **currently** affecting your running goals?

- Very concerned (1)
- Somewhat concerned (2)
- Neutral (3)
- Somewhat unconcerned (4)
- Not concerned at all (5)

Q44 Please feel free to provide additional comments about how COVID-19 has affected your training goals (i.e. race disruptions, etc.).:

________________________________________________________________

End of Block: Running behaviors during the COVID-19 pandemic

Start of Block: Injury Status During COVID-19

Q34 **During social distancing restrictions in your region due to the COVID-19 pandemic,**did you suffer from any running-related injuries?

- Yes (1)
- No (2)

Skip To: End of Block If During social distancing restrictions in your region due to the COVID-19 pandemic, did you suffer... = No

Q35 How many running-related injuries did you sustain**during social distancing restrictions in your region due to the COVID-19 pandemic?**

▼ 1 (1) ... Greater than 10 (11)

Q34 How long were you unable to perform running training due to running-related injuries sustained d**uring social distancing restrictions in your region due to the COVID-19 pandemic?**

▼ 1 day (1) ... 1-2 months (22)

Q44 How long did you have to modify your running training due to running-related injuries sustained **during social distancing restrictions in your region due to the COVID-19 pandemic?**

▼ 1 day (1) ... 11-12 months (54)

Q36 For the running-related injuries you sustained **during social distancing restrictions in your region due to the COVID-19 pandemic,** please type the number of injury types occurring at each body location in the text boxes provided.

|  | Injury Type | | | |
| --- | --- | --- | --- | --- |
|  | Sprain (ligament) (1) | Strain (muscle or tendon) (2) | Fracture (broken bone) (3) | Other (please explain) (4) |
| Toe (1) |  |  |  |  |
| Foot (10) |  |  |  |  |
| Ankle (2) |  |  |  |  |
| Lower leg (3) |  |  |  |  |
| Knee (4) |  |  |  |  |
| Thigh (5) |  |  |  |  |
| Hamstring (6) |  |  |  |  |
| Hip (7) |  |  |  |  |
| Groin (11) |  |  |  |  |
| Abdomen (8) |  |  |  |  |
| Low Back (9) |  |  |  |  |

End of Block: Injury Status During COVID-19

Start of Block: Follow-Up Survey

Q44 Would you be willing to complete a follow-up survey on your running behaviors to be emailed to you after social distancing measures are lifted?

- Yes (5)
- No (6)

Display This Question:

If Would you be willing to complete a follow-up survey on your running behaviors to be emailed to yo... = Yes

Q45 Please provide your full email address below:

________________________________________________________________

End of Block: Follow-Up Survey
