# Supplementary material for: Running behaviors, motivations, and injury risk during the COVID-19 pandemic: A survey of 1147 runners
Source: PLoS One. 2021 Feb 12;16(2):e0246300. doi: 10.1371/journal.pone.0246300 (PMC7880469; doi:10.1371/journal.pone.0246300)
Supplement: S1 Table — The words or phrases that most frequently occurred in short responses were grouped into like categories and displayed in the table, along with number of instances noted throughout the short responses. (DOCX) [file pone.0246300.s003.docx]

| “race(s)” (450) / “marathon/ half-marathon” (127) / “meets” (5) / “5-K” or other numerical distance races (28) | 610 instances |
| --- | --- |
| “cancelled” (221) / “disruptions” (51) / “closed” (38)/ “postponed” (27) / “changed” (38)/ “delayed" (6) | 381 instances |
| “motivation/motivated” (101)/ “goal(s)” (91) | 192 instances |
| “less” (47)/ “much” (52)/ “unable” (8) / “miss” (29)/ “without” (28)/ “decreased” (21) | 185 instances |
| “injury” (47) / “hurt” (3) / “pain” (5) / “health” (14) | 69 instances |
| “still” (39) / “virtual” (50) / “kept” (31) / “continue” (13) / “able” (48) | 181 instances |
| “increased” (46)/ “longer” (61) / “faster” (19)/ “shorter” (15) / “slower” (12)/ “volume” (6) / “pace”(22) | 181 instances |
| “social” (25)/ “group(s)” (54)/ “people” (29)/ “team” (8)/ “together” (5) | 121 instances |

S1 Table. Open coding short response items.
