# Supplementary material for: Running behaviors, motivations, and injury risk during the COVID-19 pandemic: A survey of 1147 runners
Source: PLoS One. 2021 Feb 12;16(2):e0246300. doi: 10.1371/journal.pone.0246300 (PMC7880469; doi:10.1371/journal.pone.0246300)
Supplement: Attachment — Submitted filename: R2_Response_to_Reviewers.docx [file pone.0246300.s005.docx]

Reviewer #1: Thank you for your revised paper and replies to my comments.	**Response: We would like to thank Reviewer 1 for taking the time to review the	revised manuscript. We addressed all specific comments below.**

A few points:
Line 10, 152, 205. Student´s t test	**Response: Paired t-tests were changed to Student’s t-tests on lines 10, 153, 166, 206,	213.**

L13, 284. Sentences should never start with a number in numerical format.	**Response: The sentence structure was adjusted on lines 13 and 284.**
L52, 165, 191, 206, 377 Something wrong with the text- part of it seems to be cut off	**Response: We apologize that the text was cut off for the uploaded version, we are	unsure what happened as we checked to make sure everything looked in order on	the submission. There does not appear to be any truncated text on this modification,	we apologize again for the error.**
L56 moulding?	**Response: We revised “molding” to “influencing” to enhance clarity.**
L73, 88, 89, 116 etc RRI´s (you left out the apostrophe)	**Response: All instances of “RRIs” have been fixed to “RRI’s”**
L130-131, 132, 196-198, 301 “participants residing in all countries”, “through June”, “about participants’ perceived impact the pandemic had on”, “participants that” are ungrammatical	**Response: We edited each sentence to be grammatically correct.**
L155 as compared to	**Response: We added “as” to the sentence on line 155.**
L178 “put into in the model” needs to be corrected	**Response: We adjusted this to now read “and put into the model” on line 179.**
Table 1 units incomplete (e.g. non for pace)	**Response: We added in all units now in Table 1.**
I think you should explicitly state that injury was not defined in the survey	**Response: We added this information on line 117.**
Thank you.

Reviewer #2: Thank you for your thorough revision of the manuscript and your honest and clear responses. Although the study protocol and collected data did not allow all concerns to be fully remediated, the authors have listed these as a study limitation and revised the manuscript and conclusions accordingly whenever possible. I think the editor is now best suited to evaluate the manuscript. If the manuscript still fits the scope and policy of Plos One, I could consent with accepting this manuscript for publication.	**Response: We would like to thank Reviewer 2 for taking the time to review the	revised manuscript, and for your feedback and comments throughout this process.**
